# Selecting criteria for the right prosthesis in defect of the abdominal wall surgery

**Published:** 2009

**Authors:** H Mohamed, D Ion, M.B. Serban, M Ciurea

**Affiliations:** * University Emergency Hospital Bucharest

## Abstract

The article is debating a theme of great interest for the defect of the abdominal wall surgery – the use of biocompatible prosthesis. The surgeon is often confused by the avalanche of offers made by the mesh producers, making it mandatory for him to know very well the behavior of these alloplastic structures in the tissue environment.

From this point of view, we have discussed both the physicochemical properties and the histological reaction brought by the most common type of meshes: polypropylene, polyethylene – tereftalat, polytetrafluorideethylene. This presentation brings out the minimal but mandatory criteria for any mesh to be accepted, but also the criteria that need to be taken into consideration when we try to improve the qualities of the mesh closer to the desideratum of the “ideal mesh”.

The main conclusion of this review is that we have to change the myth of the “ideal mesh” with “the right chosen mesh”, that based on its chemical, physical, structural and biological qualities will adapt perfectly first to the patient’s needs and second to the surgeon’s needs.

## Introduction

The inguinal hernia remains the most frequent pathology in general surgery hospitals in many countries of the world, rising both medical and socioeconomically problems. It is estimated that the rate of the disease reached 3% in the general population, rising to a 10–15% in the adult population, from which the overwhelming majority, 90%, is represented by males.

The scientific bases of the hernia surgery began in 1877 when Eduardo Bassini achieved the radical cure for the inguinal hernia by strengthening the weak inguinal area without limiting its actions only to the approach of the hernia sac, as it was done until that time.

The Bassini procedure has remained the reference guide in the inguinal hernia surgery for more than 100 years, until 1989, when L.I. Lichtenstein and colab. published a study of more than 1000 cases that underwent hernia surgery using a prosthesis made of a textile material for the strengthening of the posterior wall at the inguinal canal, with very good results.

A new era began from that point on in the approach of the inguinal hernia surgery that, in spite of the extraordinary benefits, raised a multitude of questions about finding the “right prosthesis”.

## Biomaterials

All modern materials used in surgical practice have been improperly called “plastic materials”. The concept is inappropriate because the term “plastic” does not have a chemical meaning, and it can describe, at the same time, both organic, semi organic and even organic substances. Furthermore, this term refers only to the mechanical capability and partially to the elastic one. Based on these considerations, it is more appropriate to define them as biopolymers or biomaterials (bioprosthesis).

Based on the response to heat and pressure, the biopolymers are classified into two major categories:

***1. Thermoplastic resin:*** under the action of the heat it suffers a transformation cycle entirely reversible; their shape can only be altered during successive exposure to heat. They are composed of linear macromolecules and are represented by the next classes of substance:

a) polyvinyl (polyvinyl chlorate, polyvinyl acid);

b) polyolefin (polyethylene, polypropylene);

c) fluoride polymer (polytetrafluorideethylene, polyvinyl den fluoride);

d) polyamides (nylon);

e) saturated polyesters with high molecular weight (Dacron, Rhodergon).

2. thermo labile resin: tridimensional molecules obtained by polycondensation. Under the action of heat it suffers an irreversible physico – chemical transformation followed by the loss of its plasticity. The main representatives used in surgery are epoxies, polyurethanes and silicones. 

Based on the texture, the synthetic prosthesis can be classified into two major classes:

A. meshes (braided networks);

B. plates (rarely used, barring the polytetrafluorideethylene plate – Teflon, Gore-Tex, Dual Mesh).

All meshes are based upon a polymeric filament. They are made of a created knitted network (with variable loops) that offers a maximum mechanical resistance. The knitted loops can be distributed along the longitudinal axle of the fabric (ideal because of its greater resistance), or the transversal axle. This is a typical property for the knitted textile fabric that must decide the implantation direction of the mesh, so that the direction of the physiological abdominal wall forces is parallel to the elongation of the mesh.

Based on the behavior of the prosthesis in the tissue environment, the meshes are divided into resorbable and non resorbable. Because the first category represents only a temporary support for the abdominal wall, adequate to the septic environment, it can’t be used in the definitive treatment of abdominal wall defects. The second category is divided into monofilament and multifilament. 

In fact, the most frequently used meshes, after passing the experimental and clinical trials, are:

*1. polypropylene*,

*2. polyethylene – tereftalat*,

*3. polytetrafluorideethylene*.

Starting from these three major meshes a continuous improvement of the physico-chemical qualities began, that would bring them closer and closer to the demands of the ideal prosthesis.

***1) Polypropylene***

It is a thermoplastic discovered in 1956 by Giulio Nata, while performing an izotactic polymerization of ethane, which he added a carbon – methyl complex to and transformed it into propane. The mechanical properties of polypropylene depend on the crystalline degree which reflects in its density.

The molecular weight of the polypropylene is 100.000 Da. The strength of polypropylene is similar to that of the iron, despite its density of 1/8.

Polypropylene is resistant to biological decay and relatively impermeable to water steam. Also it is not liable to enzymatic degradation *in vivo*. The structure of this type of material gives it high thermo resistance, up to 168ºC, making the sterilizing process possible without altering its properties. Polypropylene is made up of multiple series of linear long and flexible chains. The reason why this type of material is so biocompatible is that during its extraction it needs a small amount of catalysts and additives. The wire is continuous, monofilament and non resorbable.

At present there are many meshes derived from polypropylene under different forms, dimensions and textures, according to the producer and the demands.

The pore dimension and the weight of the product have significant impact on its rigidity. These factors will influence the degree of collagen infiltration on the cicatriceal tissue. 

The most common polypropylene meshes on the Romanian market are Marlex and Prolene. 

***2) Polyethylene – tereftalat***

It has been synthesized in 1941 by the English chemists Whinfield and Dickson, who combined ethylene alcohol with the thereftalic acid by polycondensation. That was the time of the emersion of “Terilene”, the first synthetic product that would replace wool and cotton. 

Despite having great mechanical resistance, the polymer has been proven by many studies to decay “in vivo”, losing its mechanical stability (decrease in diameter, decrease by 15% in rupture resistance, decrease in molecular weight and decrease by 4 – 7% in number of fibers each year). These phenomena speed up in the presence of infection.

***3) Polyester prosthesis***

This type of mesh is made up of a multifilament network which gives it great pliability and maneuverability. Mersilene is the most common and well known polyester mesh used today. It is made up of knitted and braided wires that confer the structural integrity of the fabric after cutting. One of the main advantages of Mersilene is that it can be resterilized after you open the package by conventional autoclave at 121 degrees Celsius for 20 minutes. 

Polytetrafluorideethylene is obtained by polymerization of the tetrafluorideethylene gas. Also known as Teflon, it became famous because of its proven waterproof property, as it was used for covering kitchen pots. Teflon was swiftly assimilated in the medical industry because it was chemically inert, with an extraordinary smooth surface which nothing sticks on, and so it became a potential biomaterial, useful in the defect of the abdominal wall surgery. 

The mechanical properties are: expanded polytetrafluoride-ethylene is a soft material, with a smooth surface and a micro porous structure. The multidirectional alignment of the fibers ensures an even distribution of its resistance on all plans. The chemical analysis indicates great resistance to basis, acids, heat, organic solvents or tissular fluids. It is the most widely met fluorocarboned polymer used in medicine.

In order to improve the biological properties and the incorporation of the conjunctive tissue, W.L. Gore innovated Mycro Mesh. This prosthesis has a smooth side and the other side with creeps that have at each apex macroscopic pores of approximately 1 mm in diameter each. This modification in the structure of the material helps the migration of the cells and the penetration of the micro and macro porous elements with collagen. The result is better tissular incorporation and a better and faster anchorage.

The same producer brought to the biomaterials market “Dual Mesh”, which is a type of expanded polytetrafluoride-ethylene with 2 sides. The internal side has blanks of 3 microns in order to prevent adhesion and the external side has blanks of 17 – 22 microns in order to help cellular growth and fibroblastic proliferation. This type of prosthetic material has been helping the development of the laparoscopic surgery for the ventral hernia and incision hernia from 1993. The product has developed since then by growing the fold’s dimension on the external surface, which became rugged. 

The risk of infection was diminished by incorporating antibacterial substances into the mesh (Silver and Clorhexidine). The commercial names of these products are Mycro Mesh Plus and Dual Mesh Plus.

## Selecting criteria for the mesh

It refers to rezorbability, mono or multifilament structure, strength, thickness, rigidity, porosity and biocompatibility. It is desirable for the ideal prosthetic material to be chemically inert, mechanically resistant, and physically stabile, to induce and allow tissular growth in the best conditions, not to produce or sustain infection. 

Until 1959 polyethylene prosthesis was used in most of the cases, in the defect of the abdominal wall surgery, followed by a transition to polypropylene from 1959 with the enrolment of synthetic extra materials. The evolution of these materials in the surgery of the defect of the abdominal wall has known a continuous growth, until 1989 when the usage of this prosthesis exploded, probably because of Lichtenstein’s study on the usage of the polypropylene mesh in inguinal hernia surgery.

The main role of the prosthesis is to ensure a permanent strengthening of the abdominal wall. The mesh’s resistance and elasticity must be adapted to the intra-abdominal pressure values.

The meshes are divided into absorbable or non absorbable, mono or multifilament. By far, the most important reason for choosing one mesh over another is the mechanical support it gives to the deficient abdominal wall. Under these circumstances, at present, the mesh must be non resorbable or slowly resorbable only if in time; it can be replaced by strong and efficient cicatriceal tissue.

***Resorbable meshes***

The reason why resorbable meshes such as Dexon and Vicryl do not allow a complete and efficient collagen deposit at the tissue – prosthesis interface is their rapid hydrolysis. For that, these prosthetic materials aren’t enough for solving a defect of the abdominal wall. 

***Non resorbable meshes***

The commonly used non resorbable meshes are enrolled in a long list, but the most used ones are Marlex, Prolene, Atrium, Surgipro, BioMesh (polypropylene polymer), Mersilene, Parietex, Teflon and GoreTex (expanded polytetrafluorideethylene).

***Composite prosthesis***

These types of meshes are made by combining the polymers. By doing this, the mesh is made of two components with different properties – a combination between a resorbable polymer and a non resorbable polymer. They are wrongfully called “semi resorbable” prosthesis. The resorbable component offers better flexibility and maneuverability while offering a temporary additional support. The resorbable polymer can be mixed between the non resorbable polymer fibers, (Vypro, Vypro2), or can be applied on one side of the non resorbable polymer (Parietex). The last type of mesh is commonly used for the intra peritoneal repair because of the low adherence that it offers. 

ComposixMesh is made up of a network of Marlex and a thin layer of Teflon has been enrolled on the abdominal side. It can be used to repair both primitive ventral abdominal wall defect and post operatory ventral abdominal wall defect by using open approach or laparoscopic approach. 

Parietex represents a tridimensional network of polyester fibers knitted and covered with a collagen based hydrophilic film. The resorbable collagen layer prevents the forming of intra peritoneal adherence. The product can be used both in open and laparoscopic surgery. 

***The multifilament structure***

One of the main concerns of the operating surgeon is to reduce the quantity of foreign material in the human body that can sustain or perpetuate an infection. The size of the pores and the interstice is the main characteristic that diminishes the risk of infection on the prosthesis. The key size is 10μm. Most bacteria is 1μm in diameter, whilst most macrophages and neutrophiles are larger than 10μm. Multi filament prostheses such as Mersilene, PTFE and Surgipro have interstices under 10μm. The bacteria can quarter inside these interstices, being protected from the macrophages that cannot enter these spaces. When infection occurs the mesh must be removed.

***The monofilament structure***

The ideal properties of any prosthesis are: to be inert, to be resistant to infection, molecular permeability, pliability, transparency, to fit in mechanically and biocompatibility. Polypropylene monofilament meshes are the most used type of prosthesis today in the treatment of inguinal hernia because they have most of the properties of the ideal prosthesis. The monofilament prosthesis is made by a process of knitted warping that issue a line of loops, which intersect in a zigzag pattern. This has the advantage of a superior tensional resistance and stability. The pattern of the fabric can be different from one producer to another and in terms with the surgeon’s demands.

***Tensional resistance***

The mesh must maintain its mechanical stability from the beginning and to withstand pressure up to 16N/cm2. Monofilament meshes such as Marlex, Prolene and Atrium have similar “burst” resistance, but, when the breakage occurs, it usually happens at the margins of the mesh, at the muscle – prosthesis junction. The conclusion is that more important than the mesh’s resistance is the suture’s resistance. This characteristic is defined by the mesh’s resistance to breaking from the sutures and it is measured by passing a suture wire through the mesh at 6.5mm from the edge and pulling constantly until it breaks.

***Rigidity and thickness***

A thicker and more rigid prosthesis has less transparency and a weaker interaction with the tissue beneath, which determines the mesh – tissue distance and the collagen deposit as well. Such a mesh can lead to the forming of juxtaprosthetic seroma. Fine meshes, thinner ones have the property of molding around the anatomic formations around the prosthesis, and by doing that they favor the rapid collagen depositing.

***Porosity***

The right pores dimension assures high permeability for the fibro-conjunctive penetration. This way an optimal fibrin fixation is assured in the host tissue that reduces the empty spaces between the prosthesis and the host tissue and dramatically reduces the risk of seroma. One of the main disadvantages of macro porous prosthesis is the high adhesion to the intestines. This leads to occlusion and intestinal fistulas.

Based on the porosity of the meshes, in 1997, Amid divided the most frequently used bio materials into four main categories: 

- Type 1 – total macro porous prosthesis (Atrium Compozix, Marlex, Prolene, Surgipro, Trelex). These prostheses have pores larger than 75μ in all three directions. 

- Type 2 – total micro porous prosthesis (PTFE, GoreTex, Dual Mesh). These prostheses have pores smaller than 10μ in all three directions.

- Type 3 – total macro porous prosthesis with micro porous multi filament component (Mersilene, Surgipro, Mycro Mesh).

- Type 4 – biomaterials with pores under 1 μ (Silastic, Cellgard, Dura Substitute). Because of their structure, these biomaterials cannot be used per se in the repair of the inguinal hernia, but can be associated with type 1 and 2 prostheses, as composite materials, in the intra peritoneal treatment of the inguinal hernia.

***Biocompatibility***

One of the main criteria for establishing the characteristics of the “ideal” prosthesis is not to produce adverse reactions after implanting. A good biocompatibility is necessary because these materials must resist for several decades from the moment of implantation. When the mesh is correctly placed it must not induce any allergic reactions or pain, and furthermore it must not transform the host tissue into cancer.

The differential response of the host to the prosthetic material is yet to be discovered. Mainly, there are three different types of response to a foreign material: destruction, incorporation or rejection. A “true” biocompatible prosthetic material should not induce a foreign body reaction. 

The implantation of the mesh determines an environment in which the normal components involved in the healing process are in direct contact with the foreign body. This contact is called mesh – tissue interface and represents an area of 300–600μm from the width of the implanted prosthetic surface. The order of the healing events is the same as in any wound healing, and goes as it follows: coagulation, inflammation, angiogenesis, the forming of the matrix and maturation.

## Conclusions

1. The surgeon who is called out to solve a defect of the abdominal wall is “bombarded” with a multitude of mesh offers from the profile industry which is in continuous evolution. This is why he must possess the right information that would be based on the surgical judgment, which is absolutely necessary to choose the optimal prosthesis for each case. 

2. The most important aspects of the “in vivo” behavior of the meshes are:

- all meshes have persistent inflammatory reactions for many years after the implantation;

- the tissular inflammatory reaction is in direct proportion with the weight of the prosthesis (g/m2) and with the contact surface;

- biocompatibility is primarily influenced by the physical properties of the material and secondly by the chemical components;

- compatibility grows with the lowering of the weight, the rising of the porosity and the mono filament structure of the prosthesis.

3. With time it has become more obvious that there is no “ideal” surgery for the inguinal hernia, and, in these conditions, it is hard to believe that the “ideal” prosthesis, universally adaptable, can be made. That’s why the “ideal mesh” myth must be changed with “the right chosen mesh”, that is based on its chemical, physical, structural and biological qualities which will perfectly adapt firstly to the patient’s needs and secondly to the surgeon’s needs.
